# The xeric side of the Brazilian Atlantic Forest: The forces shaping phylogeographic structure of cacti

**DOI:** 10.1002/ece3.3458

**Published:** 2017-10-04

**Authors:** Fernando Faria Franco, Cecília Leiko Jojima, Manolo Fernandez Perez, Daniela Cristina Zappi, Nigel Taylor, Evandro Marsola Moraes

**Affiliations:** ^1^ Departamento de Biologia Centro de Ciências Humanas e Biológicas Universidade Federal de São Carlos Sorocaba Brazil; ^2^ Museu Paraense Emilio Goeldi Coord. Botânica/Instituto Tecnológico Vale Belém do Pará Brazil; ^3^ Singapore Botanic Gardens Singapore Singapore

**Keywords:** ABC, cactus, discrete phylogeography, Inselbergs, *PHYC*, restinga, species tree, *trnS‐trnG*, xeric enclaves

## Abstract

In order to investigate biogeographic influences on xeric biota in the Brazilian Atlantic Forest (BAF), a biodiversity hotspot, we used a monophyletic group including three cactus taxa as a model to perform a phylogeographic study: *Cereus fernambucensis* subsp. *fernambucensis*,* C. fernambucensis* subsp. *sericifer*, and *C. insularis*. These cacti are allopatric and grow in xeric habitats along BAF, including isolated granite and gneiss rock outcrops (Inselbergs), sand dune vegetation (Restinga forest), and the rocky shore of an oceanic archipelago (islands of Fernando de Noronha). The nucleotide information from nuclear gene *phytochrome C* and plastid intergenic spacer *trnS‐trnG* was used to perform different approaches and statistical analyses, comprising population structure, demographic changes, phylogenetic relationships, and biogeographic reconstruction in both spatial and temporal scales. We recovered four allopatric population groups with highly supported branches in the phylogenetic tree with divergence initiated in the middle Pleistocene: southern distribution of *C. fernambucensis* subsp. *fernambucensis*, northern distribution of *C. fernambucensis* subsp. *fernambucensis* together with *C. insularis*, southern distribution of *C. fernambucensis* subsp. *sericifer*, and northern distribution of *C. fernambucensis* subsp. *sericifer*. Further, the results suggest that genetic diversity of population groups was strongly shaped by an initial colonization event from south to north followed by fragmentation. The phylogenetic pattern found for *C. insularis* is plausible with peripatric speciation in the archipelago of Fernando de Noronha. To explain the phylogeographic patterns, the putative effects of both climatic and sea level changes as well as neotectonic activity during the Pleistocene are discussed.

## 1 INTRODUCTION

In the recent past, the Brazilian Atlantic Forest (BAF) covered around 150 million hectares with distinct climatic conditions and over complex landscapes, occupying a wide latitudinal interval of around 30° (Ribeiro, Metzger, Martensen, Ponzoni, & Hirota, 2009). Such heterogeneous conditions favored the establishment of a biome with outstanding species richness and endemism (Myers, Mittermeier, Mittermeier, da Fonseca, & Kent, 2000). The BAF comprises two distinct bioclimatic regions (northern and southern), with a transition zone near the Doce River (Carnaval et al., 2014). Plant taxa restricted to only one of these regions are common, resulting in strong floristic distinction between the northern and southern BAF (Fiaschi & Pirani, 2009). Moreover, recent studies suggest that BAF combines influences of historical connections with other biomes such as the Amazon forest (Sobral‐Souza, Lima‐Ribeiro, & Solferini, 2015), seasonally dry tropical forest (SDTF, Mogni, Oakley, & Prado, 2015), and Cerrado (Antonelli, Verola, Parisod, & Gustafsson, 2010), resulting in a wide heterogeneity of plant communities. Although BAF harbors predominantly evergreen rainforest, it also includes xeric or open vegetation areas differing markedly from that of the surroundings, such as the open scrub vegetation along the sandy coastal plains named restinga, as well as the inselberg flora (Scarano, 2002).

Restinga communities are predominantly Quaternary habitats characterized by sandy soils mainly covered by herbaceous and shrubby xeric vegetation exposed to oceanic influence and direct solar radiation (Scarano, 2002). This vegetation extends in a narrow belt along most Brazilian coastal plains between evergreen forest and the sea. Inselbergs are isolated rock outcrops of Precambrian granite and gneiss, harboring a rich flora associated with harsh conditions such as poor soil, high temperature, and insolation, leading to low moisture retention (Porembski, 2007). As these rock outcrops are mostly embedded in a forest matrix, they are frequently considered continental islands (Pinheiro et al., 2013; Porembski, 2007).

Different factors are proposed to explain phylogeographic patterns in BAF, including rivers (e.g., Cazé et al., 2016; Neto, Furtado, Zappi, Filho, & Forzza, 2016) and geological faults (Batalha‐Filho et al., 2013; Thomé, Zamudio, Haddad, & Alexandrino, 2014; Thomé et al., 2010) as putative geographic barriers. Further, Pleistocene climatic changes have also been invoked to explain diversification within BAF (e.g., Cabanne et al., 2016; Cardoso, Cristiano, Tavares, Schubart, & Heinze, 2015), by persistence of rainforest species in stable areas (refugia) in the northern bioclimatic region and expansion of open vegetation formation in the south (Carnaval et al., 2014). These global climatic changes also had impacts on sea level, which in turn might have influenced the geographic distribution of coastal vegetation (Ramos‐Fregonezi et al., 2015), for instance by the exposition of Brazilian continental shelf during glacial periods (Leite et al., 2016).

In order to contribute to the understanding of the diversification events in xeric habitats of BAF, we performed a phylogeographic study with the columnar cacti *Cereus fernambucensis* Lemaire and *C. insularis* Hemsley (Cactaceae; Cereeae), which represents a Pleistocene monophyletic lineage (Franco et al., 2017). While *C. insularis* grows only on the small oceanic archipelago of Fernando de Noronha (3.8°S, 32°W), *C. fernambucensis* has a wider distributional range along xeric areas of BAF and is represented by two allopatric subspecies named *C. fernambucensis* subsp. *fernambucensis* and *C. fernambucensis* subsp. *sericifer* (Ritter) Taylor & Zappi (Taylor & Zappi, 2004). The main morphological distinctions between these subspecies are the size of the vegetative body and flowers, flower color, and fruit color (see Appendix S1). *Cereus fernambucensis* subsp. *fernambucensis* is a characteristic component of restinga forest, growing in dunes and rocky seashores along the eastern Brazilian coastal plains, with latitudes around 5–25°S. Conversely, *C. fernambucencis* subsp. *sericifer* has an inland and more fragmented distribution, associated with granitic and gneissic inselbergs in southeastern Brazil.

Here, we addressed several biological questions regarding the studied *Cereus* taxa and delineate the following predictions: (i) considering the close phylogenetic relationship between *C. insularis* and *C. fernambucensis* (Franco et al., 2017) and based on island biogeography assumptions (Cowie & Holland, 2006), we expect a peripatric origin of *C. insularis* caused by a founder effect from continental populations, likely leading to the paraphyly of the progenitor lineage (*C. fernambucensis* subsp. *fernambucensis*); (ii) higher population divergence in *C. fernambucensis* subsp. *sericifer* due to its more fragmented range in comparison with *C. fernambucensis* subsp. *fernambucensis*; (iii) based on previous phylogeographic data from co‐occurring restinga species of cactus (Menezes et al., 2016) and cactophilic flies (Franco & Manfrin, 2013), we expect to find population fragmentation along restinga forest; and finally, (iv) based on Pleistocene Refuges Hypothesis (PRH), it is expected that paleoclimatic Pleistocene oscillations should have influenced the demographic dynamics of our focal group in the past, likely promoting range expansion and higher genetic connectivity during glacial periods and, consequently, shaping the present‐day patterns of geographic distribution and population structure.

## 2 MATERIALS AND METHODS

### 2.1 Sampling and molecular methods

**Figure 1:**
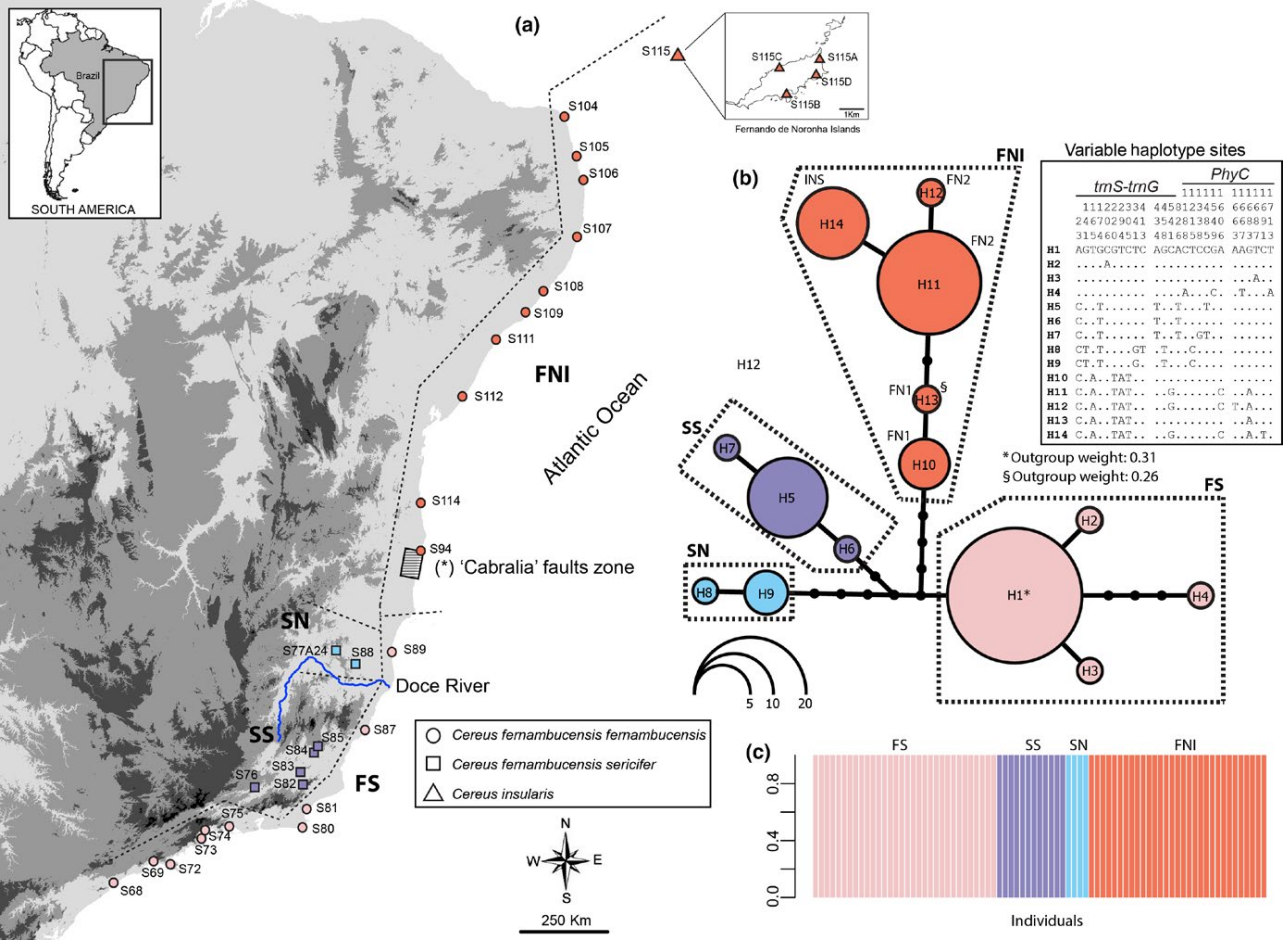
(a) Topographic map showing *Cereus* locations sampled for this study. The location codes are listed in Table 1. (b) Haplotypes network showing genealogical relationships among haplotypes of the species from *Cereus fernambucensis* subsp. *fernambucensis*,* C. fernambucensis* subsp. *sericifer*, and *C. insularis* based on the concatenated information of *trnS‐trnG* plastid intergenic spacer and *phytochrome C* nuclear gene. The size of circles is proportional to the haplotypes frequency according to the legend. Each line corresponds to one mutational step, and the small circles represent missing haplotypes. The haplotypes are colored according to the genetic population group estimated in species tree and DAPC analysis. The two haplotypes with higher outgroup weight in statistical parsimony analysis are highlighted. The variable sites among haplotypes are showed aside haplotypes network. (c) Population genetic structure inferred by DAPC analysis (*K* = 4). Each individual is represented as a vertical bar indicating its genomic composition according to each group. (*) Lima et al. (2006) described three geological faults between cities of Caraiva, BA (16.80S, 39.15W), and Santa Cruz Cabralia, BA (16.28S, 39.02W), which here we are informally named these faults as “Cabralia” faults

We sampled individuals from 31 localities, including 20 locations of *C. fernambucensis* subsp. *fernambucensis*, seven of *C. fernambucensis* subsp. *sericifer*, and four from *C. insularis*, covering the entire documented distribution of our ingroup (Table 1; Figure 1). Total Genomic DNA was extracted from root tissues with DNeasy Plant Mini Kit (Qiagen). Exon 1 from nuclear *phytochrome C* (*PHYC*) gene and the plastid intergenic spacer *trnS‐trnG* were used as molecular markers. These segments were selected based on previous variability screening for *Cereus* (Romeiro‐Brito, Moraes, Taylor, Zappi, & Franco, 2016; Silva et al., 2016). Amplification reactions for *trnS‐trnG* and *PHYC* were performed following Bonatelli, Zappi, Taylor, and Moraes (2013) and Helsen, Browne, Anderson, Verdyck, and Dongen (2009), respectively. The direct sequencing was prepared using the Big Dye terminator 3.1 kit (Applied Biosystems) and conducted in Gene Amp PCR System 9700 (Applied Biosystems). The forward and reverse sequences were assembled in Chromas 1.5 software, and the alignments were performed in ClustalW (Thompson, Higgins, & Gibson, 1994). No heterozygous site was identified for *PHYC* by considering the absence of potential double peaks after inspection of the sequencing chromatograms.

**Table 1:** Sample information of *Cereus* used in this study

Species	Geographic coordinates (S, W)	Voucher	Collection date	N1 (*trnS‐trnG*)	Accession N1	N2 (*PHYC*)	Accession N2
*Cereus fernambucensis* subsp. *fernambucensis*							
Peruíbe, SP (S68)	−24.25, −46.90	SORO 2657	13‐VII‐2011	5	KY575682–KY575686	3	KY575778–KY575780
Bertioga, SP (S69)	−23.76, −45.88	SORO 2742	14‐VII‐2011	4	KY575716–KY575719	0	—
Ubatuba, SP (S72)	−23.83, −45.42	SORO 2658	15‐VII‐2011	5	KP017430, KY575678–KY575681	5	KY575781–KY575785
Paraty‐Mirim, RJ (S73)	−23.22, −44.63	SORO 2659	16‐VII‐2011	2	KY575741–KY575742	2	KY575786–KY575787
Angra dos Reis, RJ (S74)	−23.04, −44.55	SORO 2660	16‐VII‐2011	3	KY575720–KY575722	3	KY575788–KY575790
Ilha de Itacuruçá, RJ (S75)	−22.95, −43.91	SORO 2661	17‐VII‐2011	5	KY575723–KY575727	5	KY575792–KY575796
Arraial do Cabo, RJ (S80)	−22.97, −42.03	SORO 2663	06‐X‐2011	4	KY575687–KY575690	4	KY575804–KY575807
Rio das Ostras, RJ (S81)	−22.53, −41.93	SORO 2664	07‐X‐2011	5	KY575728–KY575732	4	KY575808–KY575811
Guarapari, ES (S87)	−20.64, −40.43	SORO 2668	08‐X‐2011	4	KY575691–KY575694	4	KY575817–KY575820
São Matheus, ES (S89)	−18.76, −39.75	SORO 2669	10‐X‐2011	4	KY575695–KY575698	4	KY575825–KY575828
Cabrália, BA (S94)	−16.28, −39.02	SORO 4569	26‐VII‐2012	3	KY575763–KY575765	5	KY575829–KY575833
Maracajaú, RN (S104)	−5.39, −35.31	SORO 2670	20‐XI‐2012	2	KY575745–KY575746	2	KY575834–KY575835
Baía Formosa, RN (S105)	−6.37, −35.01	SORO 2670	20‐XI‐2012	1	KY575747	2	KY575836–KY575837
Cabedelo, PB (S106)	−6.97, −34.83	SORO 2671	20‐XI‐2012	2	KY575748–KY575749	2	KY575838–KY575839
Porto de Galinhas, PE (S107)	−8.42, −34.98	SORO 2672	21‐XI‐2012	3	KY575750–KY575752	3	KY575840–KY575842
Maceió, AL (S108)	−9.79, −35.86	SORO 2673	22‐XI‐2012	3	KY575753–KY575755	3	KY575843–KY575845
Coruripe, AL (S109)	−10.33, −36.31	SORO 2674	22‐XI‐2012	2	KY575756–KY575757	2	KY575846–KY575847
Aracaju, SE (S111)	−11.02, −37.07	SORO 2676	23‐XI‐2012	3	KY575758–KY575760	3	KY575848–KY575850
Imbassaí, BA (S112)	−12.46, −37.94	SORO 2747	23‐XI‐2012	2	KY575761–KY575762	3	KY575851–KY575853
Una, BA (S114)	−15.11, −39.00	SORO 2675	25‐XI‐2012	3	KY575766–KY575768	5	KY575854–KY575858
*Cereus fernambucensis* subsp. *sericifer*							
Três Rios, RJ (S76)	−22.01, −43.27	SORO 2662	18‐VII‐2011	7	KP017431, KY575700–KY575705	7	KY575797–KY575803
Santa Maria Madalena, RJ (S82)	−21.95, −42.03	SORO 2665	07‐X‐2011	3	KY575706–KY575708	0	—
Itaocara, RJ (S83)	−21.65, −42.09	SORO 2666	07‐X‐2011	5	KY575733–KY575737	0	—
Itaperuna, RJ (S84)	−21.22, −41.74	SORO 2667	07‐X‐2011	3	KY575738–KY575740	3	KY575812–KY575814
Bom Jesus do Norte, RJ (S85)	−21.07, −41.66	SORO 2745	08‐X‐2011	5	KY575709–KY575713	2	KY575815–KY575816
São João do Manteninhas, MG (S77A24)	−18.73, −41.19	SORO 2775	21‐IX‐2011	1	KY575777	1	KM888771
Águia Branca, ES (S88)	−19.06, −40.69	SORO 2749	09‐X‐2011	4	KY575714, KY575715, KY575743, KY575744	4	KY575821–KY575824
*Cereus insularis*							
Porto de São Pedro, F. Noronha, PE (S115A)	−3.83, −32.40	SORO 2677	10‐X‐2013	2	KY575769–KY575770	2	KM888777, KY575860
Forte do Sueste, F. Noronha, PE (S115B)	−3.87, −32.42	SORO 2677	11‐X‐2013	2	KY575771–KY575772	2	KY575861–KY575862
Praia do Boldró, F. Noronha, PE (S115C)	−3.84, −32.43	SORO 2677	11‐X‐2013	2	KY575773–KY575774	2	KY575863–KY575864
Praia do Atalaia, F. Noronha, PE (S115D)	−3.85, −32.40	SORO 2677	12‐X‐2013	2	KY575775–KY575776	2	KY575865–KY575866

N1, *trnS‐trnG* sample size. N2, *PHYC* sample size.

### 2.2 Phylogeographic analyses and population structure

The nucleotide substitution model was inferred with jModelTest (Darriba, Taboada, Doallo, & Posada, 2012), adopting the Akaike Information Criterion (AIC). The best models for *trnS‐trnG* and *PHYC* were TN93 (Tamura & Nei, 1993) and HKY (Hasegawa, Kishino, & Yano, 1985), respectively. Networks for each marker were generated in Haplotype Viewer (http://www.cibiv.at/~greg/haploviewer) assuming as input a maximum‐likelihood (ML) topology generated in Mega 5.1 (Tamura et al., 2011) and also by statistical parsimony implemented in TCS v1.21 (Clement, Posada, & Craldall, 2000). To test the phylogenetic congruence between the plastid and nuclear datasets, we perform the Congruence Among Distance Matrices test (CADM) (Campbell, Legendre, & Lapointe, 2011), as implemented in package Ape in R. The level of congruence in this analysis ranges from 0 to 1 as estimated by Kendall's coefficient of concordance (*W*) (Campbell et al., 2011).

Species tree (Edwards, Liu, & Pearl, 2007) was estimated using BEAST2 (Bouckaert et al., 2014), assuming the selected substitution model, Yule tree coalescent prior and the relaxed LogNormal clock model for *PHYC* and strict clock model for *trnS‐trnG*. The clock model for each partition was selected after comparison of the marginal likelihoods from independent runs assuming strict or relaxed lognormal clocks in a path sampling analysis with eight steps and 500 thousands generation after a 50% burn‐in. The species tree was obtained after 100 million MCMC generations, with a 25% burn‐in, and sampling trees every 5,000 steps. Divergence time for each node was estimated using a uniform prior distribution for the plastid marker *trnS‐trnG* including the minimum and maximum substitution rates observed in the chloroplast sequences of angiosperms, that is, 0.29% and 0.11% of substitution per million years (Bonatelli et al., 2014; Wolfe et al., 1987), respectively, and using a wide prior for *PHYC* evolutionary rate following Perez, Bonatelli, Moraes, and Carstens (2016).

The discriminant analysis of principal components (DAPC) was performed in the R package adegenet (Jombart, Devillard, & Balloux, 2010) after a preliminary run using the smallest number of principal components (PC) that accounted for the total variance in the data, and for a crescent number of clusters (*K*) from 2 to 10, to assess the most likely number of groups through Bayesian Information Criterion (BIC). An optimization procedure was carried to select the number of PCs, in order to maximize the successful reassignment of data, measured as the α‐score. The output of the DAPC analysis was plotted with Distruct (Rosenberg, 2004). Global and hierarchical analysis of molecular variance (AMOVA) and standard diversity indexes were conducted with the program Arlequin 3.5.2.2 (Excoffier & Lischer, 2010).

### 2.3 Demographic analyses

The expected mismatch distribution analysis under pure demographic growth (Rogers & Harpending, 1992), as well as neutrality tests [Fu's Fs (Fu, 1997) and Tajima's D (Tajima, 1989)], was performed for each marker to test the deviation from demographic equilibrium. These analyses were employed in Arlequin 3.5.2.2 (Excoffier & Lischer, 2010). We also performed extended Bayesian skyline plot (EBSP) in BEAST2 (Bouckaert et al., 2014) using *trnS‐trnG* and *PHYC* datasets, assuming the models for nucleotide evolution and molecular clock identified for each partition. The substitution rate was available only for plastid sequences. Depending on the analysis, the MCMC runs were carried for 20 to 80 million generations sampling every 2,000 steps, with a 15% burn‐in. The results of EBSP were analyzed in TRACER 1.6 (available from http://beast.bio.ed.ac.uk/Tracer).

### 2.4 Biogeographic reconstruction

**Figure 2:**
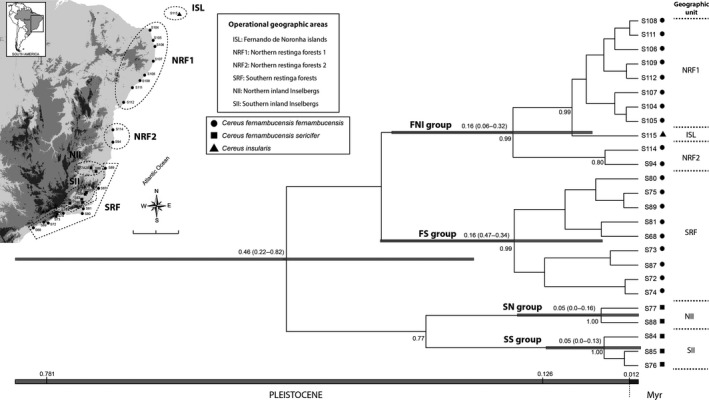
Calibrated Species Tree of *Cereus fernambucensis* and *C. insularis* locations based on *trnS‐trnG* plastid intergenic spacer and *phytochrome C* nuclear gene. Posterior probabilities are showed below branches while the estimated ages are displayed above the branches with high posterior probabilities (>0.95). The 95% HPD interval is represented in parenthesis and by the gray bars. In up left is a topographic map with the operational geographic units defined to perform our biogeographic analysis. These units were designed mainly considering the genetic differentiation among populations groups of our ingroup (see main text) together with the disjunctive distribution of *C. insularis* in Fernando de Noronha islands

To perform dispersal‐vicariance analysis (S‐DIVA) and Bayesian Binary MCMC (BBM) methods using RASP 2.0 (Yu, Harris, Blair, & He, 2015; Yu, Harris, & He, 2010), six operational geographic units (Crovello, 1981) were established based on genetic circumscription of population groups as well as considering our previous knowledge about geographic distribution, as for instance the disjunctive occurrence of *C. insularis* in a oceanic islands: Fernando de Noronha islands (ISL), southern inland Inselbergs (SII), northern inland Inselbergs (NII), southern restinga forest (SRF), northern restinga forest 1 (NRF1), and northern restinga forest 2 (NRF2) (Figure 2). We subdivided northern Atlantic coast of Brazil in two operational geographic units (NRF1 and NRF2) based on the observation that NRF1 includes internal haplotypes and is allocated in an important biogeographic region for BAF, which encompasses a unique flora (Fernandes & de Queiroz, 2015). We used 1,000 random trees from our species tree output in both analyses and assumed four as the maximum number of ancestral areas.

For discrete phylogeography (DP) approach, we implemented the diffusion model (Lemey, Rambaut, Drummond, & Suchard, 2009) in BEAST2 (Bouckaert et al., 2014) assuming the same priors of species tree analysis and the same operational geographic units as described above. The lognormal relaxed model was assumed for geographic units in this analysis, allowing variation in the diffusion process across the branches in phylogeny. We performed five independent runs of 100 million generations sampled each 5,000 steps. Log combiner v2.3.2 (Bouckaert et al., 2014) was used to combine the runs and trees after removing 15% as burn‐in. Spatial diffusion was displayed in Google Earth (https://earth.google.com/) based on the maximum clade credibility (MCC) tree using SPREAD (Bielejec, Rambaut, Suchard, & Lemey, 2011).

**Figure 3:**
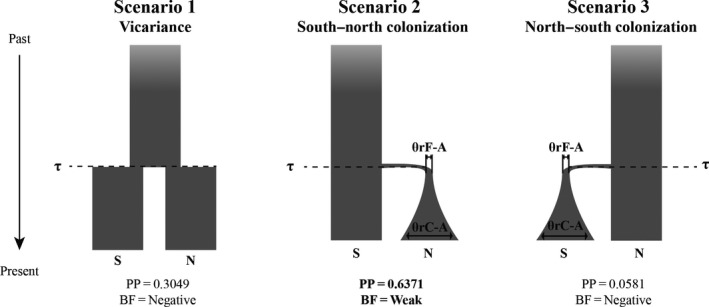
Alternative simulated models of area colonization by our ingroup in BAF used in ABC: simple vicariance (Model 1), south‐to‐north colonization (Model 2), and north‐to‐south colonization (Model 3). PP—posterior probability of each model. BF—relative Bayes Factor values of the tested model compared to the alternative model with the highest PP

Based on the results of S‐DIVA, BBM, and DP, we established three alternative scenarios of diversification to be tested with approximate Bayesian Computation (ABC). The first scenario was simple vicariance, assuming no changes in population sizes. Scenario 2 simulated south‐to‐north colonization, while scenario 3 simulated the opposite (Figure 3). Using the scripts available in Perez et al. (2016), we simulated 200 thousand datasets under each scenario, matching our empirical data. Prior values for each parameter were initially drawn from a flat uniform distribution with a wide range and then calibrated after a preliminary run. The lower and upper bounds for each parameter were selected as follows: divergence time (τ) spanning from 0.1 to 5*N*_e_ generations ago; theta values (θ) in the ancient population, ranging from 0.1 to 6 in the plastid and 0.4 to 24 in the nuclear DNA datasets. For colonization models, we sampled additional prior values related to the following: contractions in the populations during the colonization (θrF‐A), calculated as a ratio of the θ in the ancient population, sampled from an uniform distribution from 0.001 to 0.1; and the magnitude of the population expansion after the colonization (θrC‐A), calculated as a ratio of the current θ related to the ancient population, sampled from an uniform distribution from 0.01 to 1. The following summary statistics (SuSt) from empirical and simulated data were calculated according to Perez et al. (2016): proportion of polymorphic sites (π), number of segregating sites (S), Tajima's D, Fay, and Wu's θ_H_, proportion of polymorphic sites within each population (πw), and proportion of polymorphic sites between populations (πB). The performance of ABC using the original and PCA‐summarized SuSt was compared. The selected method was then used in R package ABC version 1.4 (Csilléry, François, & Blum, 2012) for model selection with a threshold level of 0.005, resulting in 3,000 simulations retained in the posterior. To assess the performance of our ABC procedure, posterior predictive checks were performed, using the estimated parameters to simulate 1,000 datasets under the best scenario.

## 3 RESULTS

### 3.1 Circumscription of genetic groups

We recovered alignments of 951 bp for *trnS‐trnG* from 101 individuals and 785 bp for *PHYC* from 89 individuals. Eight haplotypes are retrieved for *trnS‐trnG* (haplotype diversity, Hd: 0.73) and 10 haplotypes for *PHYC* (Hd: 0.74). The unique sources of sequence variation were nucleotide substitutions (see Appendix S2). As the CADM tests indicate congruence between *trnS‐trnG* and *PHYC* datasets (*W* = 0.883; *p* < 0.001), we concatenated the partitions to obtain a single network. The relationships among the resulting 14 haplotypes showed four haplogroups geographically structured: southern distribution of *C. fernambucensis* subsp. *fernambucensis* (FS); northern distribution of *C. fernambucensis* subsp. *fernambucensis* together with *C. insularis* (FNI); southern distribution of *C. fernambucensis* subsp. *sericifer* (SS); and northern distribution of *C. fernambucensis* subsp. *sericifer* (SN) (Figure 1). The DAPC analysis showed highly biased results when we used the complete dataset, including missing data. Thus, we used a subsample without missing data to perform DAPC. Preliminary runs showed that seven PCs accounted for 100% of the cumulative variance and provided three discriminant functions. The analysis of most likely number of groups based only on the BIC‐scores was uncertain, because BIC values always decreased when higher numbers of groups were tested. However, the manual inspection of group memberships indicates *K* = 4 as the highest *K*‐value without “virtual groups”, as *K* > 4 resulted in individuals with genome proportions scattered in more than one cluster. An additional DAPC run using the α‐score to refine our analysis suggests that only 3 PCs (89.6% of the total variance) needed to be retained, and we showed the results of this optimization. The four groups recovered by the DAPC matched the same grouping as revealed in the haplotypes network (Figure 1c).

The four population groups recovered in DAPC were also highly supported by the species tree analysis, but the genealogical relationships among them showed low support (Figure 2). AMOVA results were congruent with DAPC and the species tree, as the among‐group variance taking into account four genetic groups was higher than the taxonomic circumscription (Table 2). The standard indices of diversity by population group are given in Table 3.

**Table 2:** Analysis of molecular variance (AMOVA) based on *trnS‐trnG* and *PHYC* variation

Source of variation	*df*	Variance components	Percentage of variation	Fixation indices
*trnS‐trnG*				
Global AMOVA				
Among population	27	2.10022 Va	99.48	*F*_ST_: 0.99*
Within populations	73	0.01097 Vb	0.52	
Total	100	2.11119	100	
Three groups defined by taxonomic circumscriptions				
Among groups	2	1.54452 Va	55.29	*F*_CT_: 0.55*
Among population within groups	25	1.23779 Vb	44.31	*F*_SC_: 0.99*
Within populations	73	0.01097 Vc	0.39	*F*_ST_: 0.99*
Total	100	2.79328	100	
Four groups according DAPC and species tree analyses				
Among groups	3	2.86061 Va	97.37	*F*_CT_: 0.97*
Among population	24	0.06639 Vb	2.26	*F*_SC_: 0.85*
Within populations	73	0.01097 Vc	0.37	*F*_ST_: 0.99*
Total	100	2.93796	100	
*PHYC*				
Global AMOVA				
Among population	24	0.66833 Va	64.98	*F*_ST_: 0.65*
Within populations	65	0.36025 Vb	35.02	
Total	89	1.02857	100	
Three groups defined by taxonomic circumscriptions				
Among groups	2	0.48722 Va	37.67	*F*_CT_: 0.37*
Among population within groups	22	0.44588 Vb	34.47	*F*_SC_: 0.55*
Within populations	65	0.36025 Vc	27.85	*F*_ST_: 0.72*
Total	89	1.29334	100	
Four groups according DAPC and species tree analyses				
Among groups	3	0.66050 Va	53.33	*F*_CT_: 0.53*
Among population	21	0.21778 Vb	17.58	*F*_SC_: 0.37*
Within populations	65	0.36025 Vc	29.09	*F*_ST_: 0.71*
Total	89	1.23852	100	

*p‐value values lesser than .01.

**Table 3:** Standard diversity indices, neutrality tests, and mismatch distribution

Population group	Diversity indexes					Neutrality tests		Mismatch distribution	
	*N*	h	S	Hd	π	Tajima's D	Fu's Fs	Curve	SSD (*p*‐value)
*PhyC*									
FS	34	3	5	0.16	0.0004	−2.00*	−0.86	Unimodal	0.007 (.20)
FNI	38	5	4	0.63	0.0013	−2.65*	1.66	a	a
SS	12	3	2	0.32	0.0004	−1.45	−1.32	a	a
SN	5	1	0	0.00	0.0000	—	—	—	—
Total sample	89	10	12	0.73	0.0020	−0.92	−2.02	Bimodal	0.005 (.68)
*trnS‐trnG*									
FS	41	2	1	0.05	0.0001	−1.12	−1.47		0.007 (.99)
FNI	32	2	1	0.32	0.0003	0.40	0.83	a	a
SS	23	1	0	0.00	0.0000	—	—	—	—
SN	5	2	1	0.40	0.0004	−0.81	0.09	a	a
Total sample	101	8	14	0.73	0.0042	1.37	5.32	Multimodal	0.077 (.10)

*N*, number of sequences; *h*, number of haplotypes; *S*, polymorphic sites; *H*d, haplotype diversity; π, nucleotide diversity; SSD, sum of square deviation test. The codes for populations groups are the same used in Figure 1.

*p < .05.

a The least‐squares procedure to fit model mismatch distribution and observed distribution did not converge after 2,000 steps.

### 3.2 Demographic analyses

We considered the reliability of inferred demographic events according to the congruence detected by both analyzed markers. Thus, only FS group showed a signature of population expansion as evidenced by a unimodal mismatch distribution and significant negative values of Tajima's D (Table 3). However, the EBSP performed for this group did not reach convergence even after several attempts with different priors, probably due to low intragroup genetic resolution. To overcome the lack of genetic variation, we also reported the neutrality tests (Table 3) and EBSP for total sample, as we adopted a sampling strategy similar to the “pooled” scheme simulated by Heller, Chikhi, and Siegismund (2013) which minimizes the effects of substructure in demographic inferences. At any rate, we cannot reject constant population size as the parameter “number of population changes” statistically did not differ from zero [mean: 1.02 (95% HPD: 0.00–3.00)].

### 3.3 Time estimates and biogeographic analysis

The beginning of the diversification of our ingroup was recovered in the middle Pleistocene [0.46 Ma (95% HPD: 0.22–0.82 Ma)] while the crown age of each population group was estimated around middle‐to‐upper Pleistocene (Figure 2). The biogeographic reconstruction in S‐DIVA and BBM provided somewhat concordant results. Both analysis recovered the same ancestral range for the main branches and indicated dispersion followed by fragmentation from the area currently occupied by northern populations of *C. fernambucensis* to the islands of Fernando de Noronha, where *C. insularis* occurs (Table 4). The S‐DIVA recovered a large geographic area (NII + SII + SRF + NRF1) as a most probable ancestral range. For BBM analysis, the results were partially congruent (Table 4; see Appendix S3).

**Table 4:** Results of biogeographic reconstructions in S‐Diva and BBM. The geographic areas (ISL, NRF1, NRF2, SRF, NII, and SII) are described in Figure 2

Lineages	Ancestral range (probability value)^a^		Biogeographic event (probability value)^a^	
	S‐DIVA results	BBM results^b^	S‐DIVA results	BBM results
FNI	RF1 + ISL (88.27)	NRF2 (67.81), ISL (11.66), NRF1 (9.87)	Dispersion from NRF1 + NRF2 to ISL followed by vicariance between NRF2 and NRF1 + INS (*p* = .88)	Dispersion from NRF2 to ISL followed by vicariance (*p* = .26)
FN^c^	RF1 + ISL (100.00)	ISL (47.30), NRF1 (35.94), NRF2 (8.32)	Vicariance between NRF2 and INS (*p* = 1.0)	Dispersion from NRF1 to ISL followed by vicariance (*p* = .46)
FS	SRF (100.00)	SRF (99.33)	—	—
SS	NII (100.00)	NII (99.45)	—	—
SN	NRF2 (100.00)	NRF2 (97.30)	—	—
Total ingroup	SRF + NII + SII + NRF2 (35.51)	SRF (32.71), NII (25.90), SII (25.80)^d^	—	—

a Only probabilities higher than 25% are showed.

b Assuming null distribution for outgroup.

c Populations from FNI group with exception of samples from area NRF2.

d Considering wide distribution for outgroup the root area was inconclusive (see Appendix S3).

Although both S‐DIVA and BBM analyses are more suitable for higher phylogenetic levels and polytomies, as we found here, may obscure ancestral reconstructions (Yu et al., 2015) we performed the discrete phylogeography diffusion (Lemey et al., 2009) in BEAST2 (Bouckaert et al., 2014) as an additional biogeographic analysis. Despite of genetic origins remain elusive using this analysis, as all the operational units showed similar posterior probabilities to be the root area (SS: 0.19%, SN: 0.19; FS: 0.19%, FN1: 0.16%, FN2: 0.14%, INS: 0.12%), the visualization of MCT of spatio‐temporal diffusion displayed in software SPREAD corroborates the dispersal from south to the north of *C. fernambucensis* distribution, followed by colonization of the islands of Fernando de Noronha (see Appendix S4). In contrast with S‐DIVA and BBM, which allow combining the operational units, in this approach, the root area becomes restrict to one of the predefined operational geographic units. This fact could explain the lack of high support found for any of our predefined operational units.

In the face of these limitations, we used the results of previous analyses to delineate three alternative scenarios to be tested using ABC (Figure 3). The cross‐validation tests showed that summarizing the SuSt information using 7 PCA axes (90% of the variation contained in the SuSt) resulted in more accurate results compared to the original SuSt to recover the correct model (data not shown). Using this approach with the empirical data, we found that the most likely scenario (PP = 0.6371) consists of the group of southern populations founding the northern distribution (Model 2, Figure 3). Nonetheless, Bayes Factor showed low support (BF = 2.0898) for the preferred model over the simple vicariance model (Figure 3). Moreover, the posterior predictive checks also suggested an acceptable fit of our simulations to the empirical data, as all seven tested summary statistics rendered simulated datasets containing the empirical values within its 95% CI (results not shown).

## 4 DISCUSSION

The levels of genetic diversity found in both the *trnS‐trnG* and *PHYC* were similar, with the plastid marker exhibiting a higher geographic structure (Appendix S2). In plants, contrasting population structure estimates from plastid and nuclear markers might be associated with differences between seed and pollen dispersal leading to cytonuclear discordance (Petit & Excoffier, 2009). For *C. fernambucensis*, xenogamy is the predominant system of reproduction and its pollination is promoted mainly by the hawkmoth *Cocytius antaeus* (Locatelli & Machado, 1999). As the hawkmoths travel long distances (Locatelli & Machado, 1999), they may potentially spread pollen and genetic information through time among populations.

Regarding seed dispersal, the genus *Cereus* has zoochorous fruits for which dispersal is attributed to frugivorous bats, small mammals, and birds (Taylor & Zappi, 2004). There is no specific information for our focal group, but birds seem to be the main agents responsible for seed dispersal in *C. jamacaru* (Gomes, Quirino, & Araujo, 2014), a member of the sister lineage of our ingroup (Franco et al., 2017). BAF includes a high bird diversity (Myers et al., 2000); therefore, we may hypothesize that seed dispersal for cacti is also very effective in this biome. In the face of potentially high capacities for seed and pollen dispersal in our target species, the different levels of phylogeographic structure between *trnS*‐*trnG* and *PHYC* should be better explained by distinct effective population size of these markers, leading the nucleus to retain shared polymorphism for a longer time than the plastid.

Instead of a clear reciprocally monophyletic pattern for the three taxa included in our sample, we found four supported genetic groups with unresolved relationships among them. The divergence observed among these lineages, rather than the lack of phylogenetic signal, is likely explained by a rapid process of population diversification in the Pleistocene (Figure 2). Moreover, the absence of a clear signature of recent demographic shift suggests that patterns of genetic diversity in these groups were strongly shaped by the initial colonization event followed by fragmentation. Interestingly, *C. insularis* composes a monophyletic group together with northern populations of *C. fernambucensis* subsp. *fernambucensis*, leading *C. fernambucensis* to be paraphyletic (Figure 2). This is likely a consequence of accelerated population differentiation of the *C. insularis* lineage after the colonization at Fernando de Noronha by archaic continental populations of *C. fernambucensis* lineage, favoring peripatric speciation. In fact, in a peripatric model of speciation, the parental widespread lineage becomes paraphyletic (Rieseberg & Brouillet, 1994), at least until the lineage sorting renders the parental species a monophyletic status (Funk & Omland, 2003). In agreeing with the idea of its peripatric origin, *C. insularis* shown a series of morphological vegetative differences in relation to *C. fernambucensis*, including higher rib number and smaller flower size (Taylor & Zappi, 2004) that might be a consequence of founder‐event speciation which lead to rapid morphological differentiation of the peripheric and small population.

As these kind of oceanic islands were never totally connected to the continent (Cowie & Holland, 2006), peripatric speciation is far more plausible for *C. insularis*. Further, even though Fernando de Noronha orogenesis initiate during the Miocene (Cordani, 1970; Lopes & Ulbrich, 2016), it is also plausible that the long‐term colonization of this archipelago occurred during the Pleistocene, as suggested by our age estimative (Figure 2). This is justified by the occurrence of intense volcanic activity until late Pleistocene (Cordani, 1970) that must have promoted recurrent extinctions and precluded the maintenance of terrestrial biota in these islands. The Fernando de Noronha archipelago is nowadays located at about 350 km from the continent (Figure 1) as the easternmost of a chain of seamounts aligned in an east–west direction to the Brazilian continental shelf (Vital, 2014). In this chain, only the Fernando de Noronha and Atol das Rocas archipelagos are emergent nowadays. However, sea level fluctuations probably promoted massive changes in the extension of oceanic island areas, which might have affected conditions for immigration, speciation, and extinction in such islands (Weigelt, Steinbauer, Cabral, & Kreft, 2016). In glacial periods of the Pleistocene, the average sea level was much lower than at present, exposing the northeast Brazilian continental shelf, which has around 40 km of extension in this region (Vital, 2014), and probably reducing the linear distance between Fernando de Noronha and the continent. More meaningfully, the lowering of sea level exposed some of the present‐day submerged seamounts, which might have allowed a stepping stone colonization that reached Fernando de Noronha islands during the Pleistocene. A similar kind of colonization was also proposed for other oceanic islands, such as those from the Macaronesian archipelago (Rijsdijk et al., 2014).

Based on our population grouping inferences, and assuming that *C. fernambucensis* subsp. *sericifer* could be a monophyletic lineage, despite the low support in species tree for SS and SN population groups (PP = 0.77; Figure 2), we conjecture at least three main geographic centers of diversification for these cacti in BAF: (i) the inland rock outcrops and inselbergs; (ii) the southern restinga forest; and (iii) the northern restinga forest together with the islands of Fernando de Noronha. As the diversification was estimated to be within the Pleistocene (Figure 2), the influence of range shifts of BAF vegetation during this period could be hypothesized as the major driver of population differentiation across these areas, following PRH.

For BAF, majority of the evidences suggest that northern evergreen forest was more stable across Pleistocene climate changes, while the southern forest was covered predominantly by open vegetation during glacial times (Behling, 2002; Carnaval et al., 2014). Expansion of open vegetation in the past likely promoted a higher connectivity between inselberg and southern restinga vegetation. The initial fragmentation of these areas was likely driven by the isolation of inselberg and restinga biotas by subsequent expansion of the rainforest matrix between them, with dendritic infiltration along river valleys and surrounding rock outcrops during the past interglacial times. This probably explains both the initial differentiation of the *C. fernambucensis* subspecies, as well as the discontinuity in the distribution and differentiation of SN and SS populations groups of *C. fernambucensis* subsp. *sericifer*, which nowadays occur in distinct mountain ranges, separated by lowlands and river valleys (Figure 1).

Although some studies show long‐term connectivity in taxa from restinga, such as orchids (Pinheiro et al., 2011) and ants (Cardoso et al., 2015), we detected a break in geographic distribution of populations around latitude −17°, in the region politically known as southern Bahia, limiting the FS and FNI populations groups (Figure 1). Despite this area being near to the delta of the Jequitinhonha River, which has been proposed as a riverine barrier for many BAF groups, including cacti from *Pilosocereus arrabidae* (Lem.) Byles & Rowley group (Menezes et al., 2016), the FNI group includes locations on both sides of this river (the S94 location at south and the remaining at north) suggesting that Jequitinhonha river has, at least nowadays, limited impact as barrier to gene flow in *C. fernambucensis* subsp. *fernambucensis*.

To conjecture about an alternative hypothesis to explain the disjunction observed in our target species in southern Bahia, it is important to observe that this region has a unique flora (Fernandes & de Queiroz, 2015). Further, it is highlighted in several biogeographic studies as a place of disjunction (Cazé et al., 2016; Menezes et al., 2016; Pinheiro et al., 2013) or secondary contact (Carnaval, Hickerson, Haddad, Rodrigues, & Moritz, 2009; Franco & Manfrin, 2013; Pellegrino, Rodrigues, Harris, Yonenaga‐Yassuda, & Sites, 2011). As these studies include different organisms with distinct dispersal capacities, such as flies (Franco & Manfrin, 2013), amphibians (Carnaval et al., 2009), lizards (Pellegrino et al., 2011), and plants (Cazé et al., 2016; Pinheiro et al., 2013), it is reasonable that asynchrony and recurrent events such as the climatic Pleistocene oscillations could explain some of these empirical observations. However, is also possible that only PRH is an insufficient model to explain all these biogeographic data obtained from groups that diversified in different time scales and in some cases predating the Pleistocene (Pellegrino et al., 2011).

An alternative hypothesis to explain the disjunctions in southern *Bahia* is the recent tectonic activity in this region. The geological faults identified in the “Barreiras” formation from southern *Bahia*, informally named here as “Cabralia” faults (Figure 1), were likely active during the Quaternary, changing the landscape and the drainage system from a dendritic to a subparallel system (Lima, Vilas Boas, & Bezerra, 2006). Thus, before the neotectonic activity, a net of interlaced channels forming temporary coastal lagoons probably predominated (Lima et al., 2006), and those might have acted as a barrier for cacti and, together with climatic change during the Pleistocene, could have contributed to the diversification of the two population groups of *C. fernambucensis*. Likewise, these events could be related to the continuity/discontinuity dynamic inferred for other BAF taxa during the Pleistocene. Despite the fact that neotectonic activity in the “Guapiara” fault has been invoked as the main cause of vicariance in frogs (Thomé et al., 2010, 2014) and birds (Batalha‐Filho et al., 2013) in southern BAF, to the best of our knowledge, this is the first time that such activity is used to conjecture about biogeographic scenarios in northern BAF. Further studies are needed to understand the causal impact of possible tectonic activity in the “Barreiras” formation for geographic distributions of BAF biota.

The ABC results were conclusive in rejecting the north‐to‐south colonization, as model 3 showed very low PP (Figure 3), and extremely low Bayes Factor values when compared to the other two models (results not shown). Further, the independent analyses performed here indicate that south‐to‐north colonization is more likely to explain our data, despite the lack of strong statistical support to discriminate between models 1 and 2. First, a south‐to‐north colonization is congruent with all independent biogeographic reconstructions based on distinctive assumptions. Second, the internal H1 haplotype found in SFR operational unit showed the higher outgroup weight (0.31) in statistical parsimony analysis, which is consistent with a condition of an ancient haplotype. Likewise, the haplotypes from the FNI population group found in operational geographic units NRF1 (H11 and H12) and ISL (H14) present a tip position in genealogy (Figure 1) in agreement with more recent haplotypes.

The pattern of south‐to‐north dispersion for taxa associated with xeric habitats and the opposite pattern for those taxa associated with forested areas might be a widespread phylogeographic pattern in BAF, considering the idea that the northern BAF has been more stable in maintaining the forests during glaciations than the southern BAF (Carnaval & Moritz, 2008; Carnaval et al., 2014). However, this expectation is not always observed in empirical studies, probably due to the differential impacts of climatic changes in different organisms as well as other biogeographic influences and idiosyncrasies.

BAF presents complex topography, broad latitudinal variation, and sea influence leading to a miscellany of historical events that can be ascribed to explain this hyperdiverse biome (Amaral et al., 2016; Cabanne et al., 2016). However, much emphasis has been given to PRH and riverine barriers, while alternative explanations such as the role of both recent orogenic activity and regression/transgression of sea level are relatively neglected. The emphasis on riverine and PRH hypotheses may partially be attributed to the scarcity of precise geological data for most of the Brazilian coast (Thomé et al., 2010). Another possible reason is the strong tendency of researchers to explain historical events occurring during the Quaternary as a single consequence of refuge models or, particularly for BAF, to use the traditional riverine hypothesis to explain discontinuity in geographic distribution. Evidently, these are meritorious explanatory models with much support by several studies. However, in the statistical phylogeography era, these preconceptions may result in the establishment of biased and simplistic scenarios to be tested in model‐based analysis, thus limiting the emergence of new hypotheses.

The discussions about the main drivers of diversification in BAF have puzzled scientists over several years and remain controversial. In recent years, many efforts have been made to understand its astonishing diversity, including several case studies and the establishment of alternative biogeographic hypotheses, such as the putative impact of Brazilian shelf topography in the retention of forested areas (Leite et al., 2016; but see Amaral et al., 2016). Despite of this, data from taxa associated with xeric habitats in BAF are still scarce in comparison with taxa inhabiting core evergreen BAF forest. For a more complete picture of BAF biogeography, this bias needs to be minimized, as climatic events may impact taxa from forested and open vegetation areas in different ways. Furthermore, the idiosyncrasies of a particular taxon in response to climatic changes or putative geographic barriers have to be taken into account.

## CONFLICT OF INTEREST

None declared.

## AUTHOR CONTRIBUTIONS

F.F.F designed the study, samples collection, and led both the analyses and manuscript writing, which was approved by all authors. C.L.J. collected both samples and genetic data and performed several of the initial research and literature survey. M.F.P. performed species tree, DAPC, and ABC analyses and also contribute for writing. D.C.Z and N.T provided contributions in data interpretation as well as in manuscript revisions. E.M.M. helped in initial conceptions of this study, data interpretation, and writing.
