# Supplementary material for: The xeric side of the Brazilian Atlantic Forest: The forces shaping phylogeographic structure of cacti
Source: Ecol Evol. 2017 Oct 4;7(22):9281–93. doi: 10.1002/ece3.3458 (PMC5696397; doi:10.1002/ece3.3458)

*Ecology and Evolution*

**SUPPORTING INFORMATION**

**The xeric side of the Brazilian Atlantic Forest: the forces shaping phylogeographic structure of cacti**

Fernando Faria Franco, Cecília Leiko Jojima, Manolo Fernandez Perez, Daniela Cristina Zappi, Nigel Taylor and Evandro Marsola Moraes

**Appendix S1.** Natural habitat of *Cereus* species used in this study. The location codes are presented in Table 1. **(a)** *Cereus fernambucensis* subsp. *fernambucensis* (S107). **(b)** *C. fernambucensis* subsp. *fernambucensis* (S106). **(c)** *C. fernambucensis* subsp. *sericifer* (S88). **(d)** *C. fernambucensis* subsp. *sericifer* (S85). **(e)** *C. insularis* (S115B). **(f)** Volcanic rocks in Fernando de Noronha islands, Brazil, with occurrence of *C. insularis* (S115A). **(g)** Restinga forest at Ubatuba, SP, Brazil (S72). **(h)** Inselberg at region of Aguia Branca, ES, Brazil (S88).


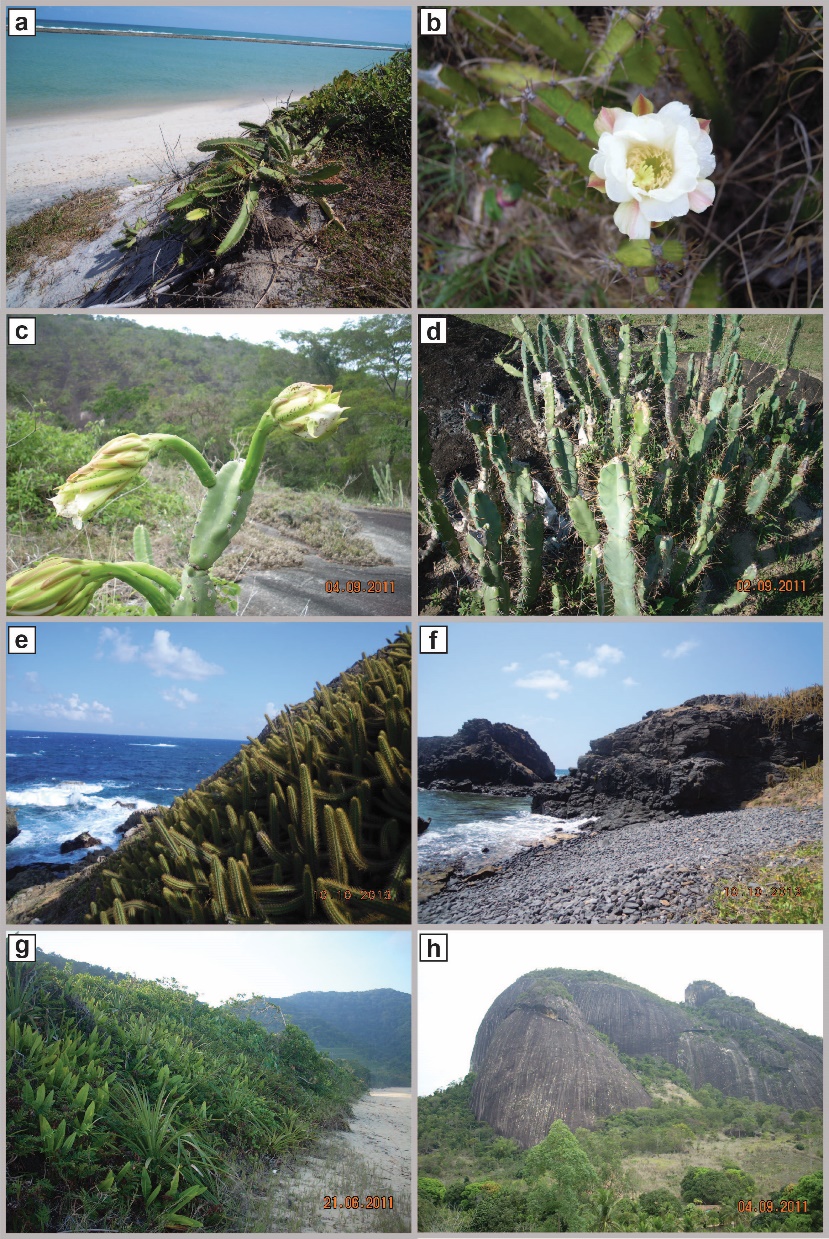


**Appendix S2.** Haplotype network showing genealogical relationships among haplotypes of the species from *Cereus fernambucensis* subsp. *fernambucensis*, *C. fernambucensis* subsp. *sericifer* and *C. insularis* based on the *trnS-trnG* plastid intergenic spacer (a) and *phytochrome C* nuclear gene (b). The haplotypes are colored according to the genetic population group estimated in species tree and DAPC analysis. The size of the circles is proportional to the haplotype frequency shown in the legend. Each line corresponds to one mutational step and the small circles represent missing haplotypes. The variable sites among haplotypes are showed aside haplotypes network.


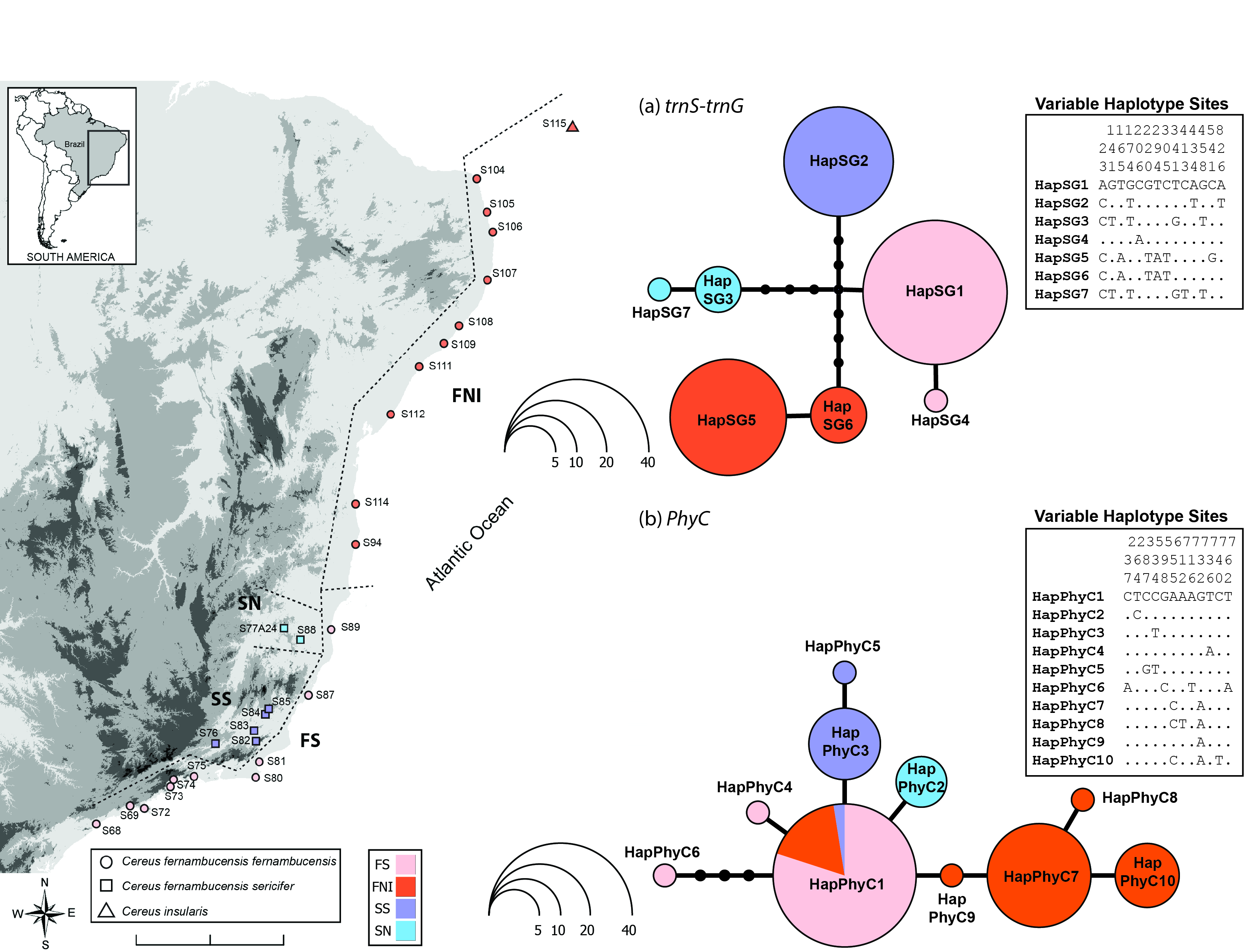


**Appendix S3**. Ancestral geographic reconstruction of *Cereus fernambucensis* + *C. insularis* branch obtained with Dispersal-Vicariance (S-DIVA) and Bayesian Binary MCMC (BBM) analyses. The results are shown only to highly supported node (posterior probability > 0.95) in species tree phylogenetic analysis. Pie charts at internal nodes represent the calculated probabilities (relative frequencies) of alternative ancestral areas, according to the colors in the legend.


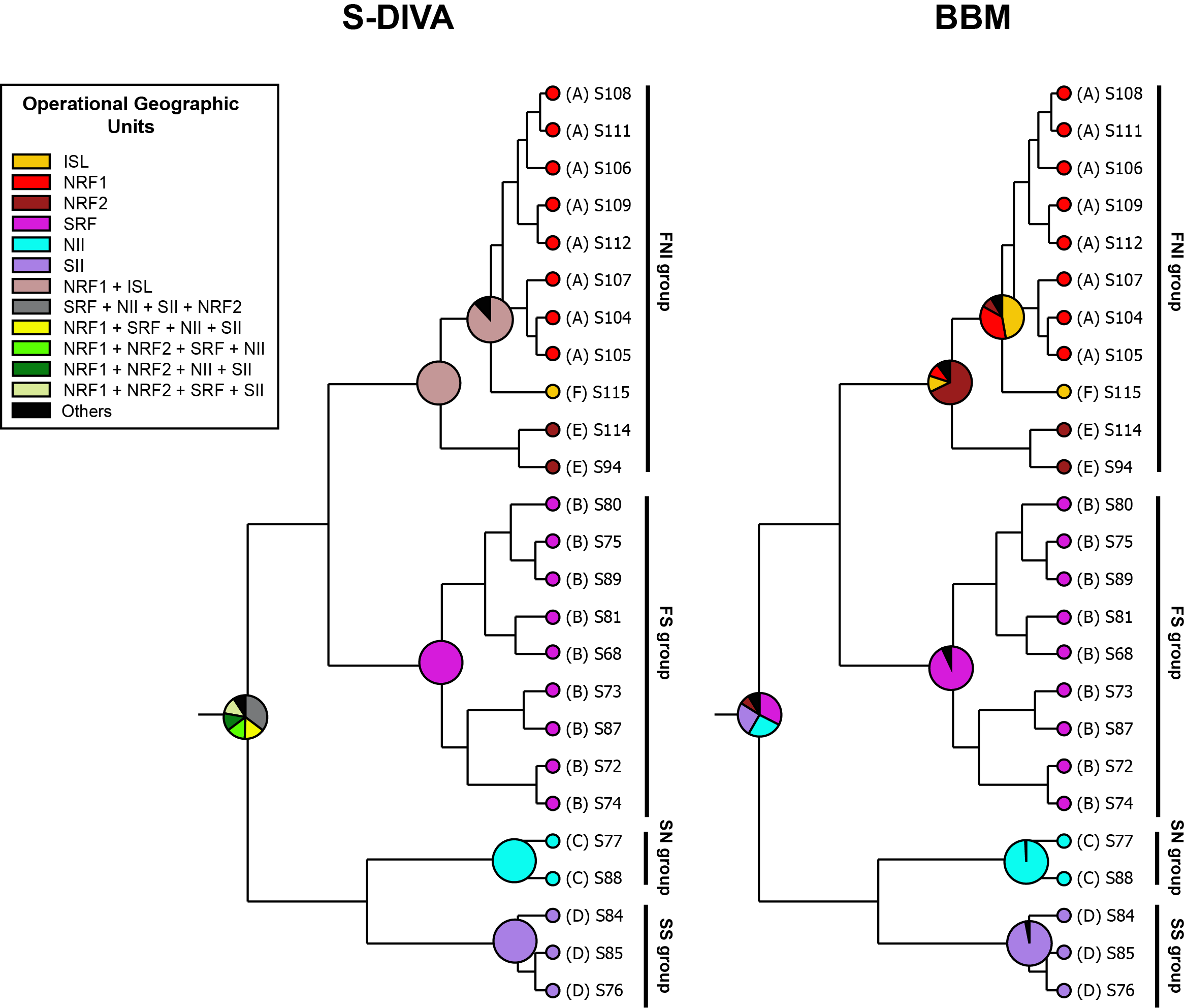


**Appendix S4**. Bayesian spatiotemporal diffusion at two time slices, based on Maximum clade credibility (MCC) tree of the clade *C. fernambucensis* + *C. insularis* estimated using Discrete Phylogeography Analysis. We assumed the central geographic coordinates of each group to reconstruct this diffusion using SPREAD software and Google Earth to visualize the output. The colored circles indicate the 95% High Posterior Density for nodes confidence intervals.


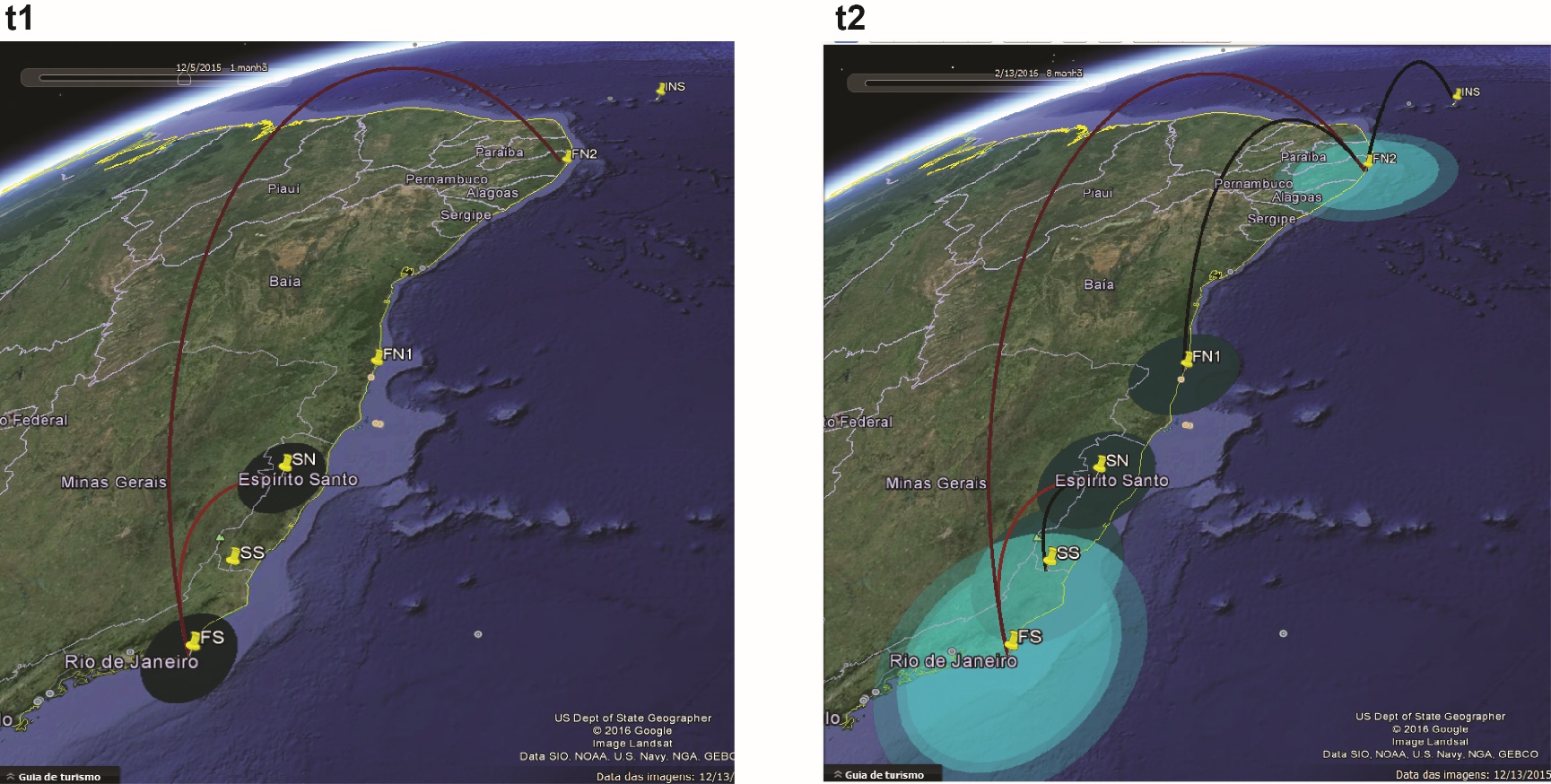

Supplement: Supplementary file 1 [file ECE3-7-9281-s001.docx]
